# LC-HRMS Profiling and Antidiabetic, Anticholinergic, and Antioxidant Activities of Aerial Parts of Kınkor (*Ferulago stellata*)

**DOI:** 10.3390/molecules26092469

**Published:** 2021-04-23

**Authors:** Hatice Kızıltaş, Zeynebe Bingol, Ahmet Ceyhan Gören, Leyla Polat Kose, Lokman Durmaz, Fevzi Topal, Saleh H. Alwasel, İlhami Gulcin

**Affiliations:** 1 Department of Pharmacy Services, Vocational School of Health Services, Van Yuzuncu Yil University, 65080 Van, Turkey; haticeakmans@hotmail.com; 2 Department of Chemistry, Faculty of Science, Ataturk University, 25240 Erzurum, Turkey; zeynep.bingol196@gmail.com; 3 Department of Medical Services and Techniques, Tokat Vocational School of Health Services, Gaziosmanpasa University, 60250 Tokat, Turkey; 4 Department of Analytical Chemistry, Faculty of Pharmacy, Bezmialem Vakif University, 34093 Istanbul, Turkey; acgoren@bezmialem.edu.tr; 5 Drug Application and Research Center, Bezmialem Vakif University, 34093 Istanbul, Turkey; 6 Department of Pharmacy Services, Vocational School, Beykent University, 34500 Buyukcekmece, Turkey; lylpolat@atauni.edu.tr; 7 Department of Medical Services and Technology, Cayirli Vocational School, Erzincan Binali Yildirim University, 24500 Cayirli, Turkey; lokmandurmaz25@gmail.com; 8 Department of Chemical and Chemical Processing Technologies, Gumushane Vocational School, Gumushane University, 29000 Gumushane, Turkey; ftopal@gumushane.edu.tr; 9 Department of Zoology, College of Science, King Saud University, Riyadh 11451, Saudi Arabia; salwasel@ksu.edu.sa

**Keywords:** *Ferulago stellata*, kınkor, acetylcholinesterase, antioxidant activity, α-glycosidase, α-amylase, polyphenol content, LC-HRMS

## Abstract

Kınkor (*Ferulago stellata*) is Turkish medicinal plant species and used in folk medicine against some diseases. As far as we know, the data are not available on the biological activities and chemical composition of this medicinal plant. In this study, the phytochemical composition; some metabolic enzyme inhibition; and antidiabetic, anticholinergic, and antioxidant activities of this plant were assessed. In order to evaluate the antioxidant activity of evaporated ethanolic extract (EEFS) and lyophilized water extract (WEFS) of kınkor (*Ferulago stellata*), some putative antioxidant methods such as DPPH· scavenging activity, ABTS^•+^ scavenging activity, ferric ions (Fe^3+^) reduction method, cupric ions (Cu^2+^) reducing capacity, and ferrous ions (Fe^2+^)-binding activities were separately performed. Furthermore, ascorbic acid, BHT, and α-tocopherol were used as the standard compounds. Additionally, the main phenolic compounds that are responsible for antioxidant abilities of ethanol and water extracts of kınkor (*Ferulago stellata*) were determined by liquid chromatography-high-resolution mass spectrometry (LC-HRMS). Ethanol and water extracts of kınkor (*Ferulago stellata*) demonstrated effective antioxidant abilities when compared to standards. Moreover, ethanol extract of kınkor (*Ferulago stellata*) demonstrated IC_50_ values of 1.772 μg/mL against acetylcholinesterase (AChE), 33.56 ± 2.96 μg/mL against α-glycosidase, and 0.639 μg/mL against α-amylase enzyme respectively.

## 1. Introduction

The plant kingdom is well known as a prolific and productive haven of phytochemicals with unmatched therapeutic potential. Moreover, 28,000 known plant taxa have been reported worldwide to have medicinal values. It has been reported that more than 3000 species have ethnomedical usage and applications against many diseases including cancer and diabetes mellitus [1]. However, herbal and medicinal plants play crucial role in the development of modern medicine and healthcare because they cause milder adverse health effects than conventional medicines and drugs [2]. According to international studies, the use of endemic and medicinal plants in the pharmaceutical, food, and cosmetic industries is constantly increasing. Meanwhile, the majority of the world’s population uses herbal medicine for basic and daily health care [3]. Medicinal plants are an important source of nutrients and secondary metabolites to protect human health. They are commonly used in developing countries and around the world, to treat some diseases especially in metabolic syndrome and diabetes mellitus [4]. It was reported that medicinal plants have many important pharmacological effects including antioxidant, anti-inflammatory, anticancer, and others. It is known that these plants have antioxidant effects and serve as sources of phenolic compounds [5,6].

Reactive oxygen species (ROS) such as singlet oxygen (^1^O_2_), hydrogen peroxide (H_2_O_2_), hydroxyl radicals (∙OH), and superoxide anion radicals (O_2_∙^−^) can easily occur as a result of normal aerobic metabolism, toxic agents, drugs, smoking, and burnt food [7,8]. The presence of ROS in the human body is very harmful due to their damage to structure and function of many biomolecules like DNA, lipid, nucleic acid, protein, and carbohydrates [9,10]. Antioxidants can easily react with free radicals or ROS and minimize damages. In addition, by slowing down or preventing its oxidation completely, they terminate radical chain reactions and minimize their harmful effects on the body metabolism [11,12]. They are synthetic or natural substances that inhibit oxidation procedure, which produce free radicals and ROS [13,14]. They can preserve the human body from these undesired effects of ROS and oxidative stress [15,16]. Antioxidants have beneficial effects in preventing chronic diseases like cancer, cardiovascular diseases, and diabetes. They prevent the occurrence of oxidative stress in humans. They can easily terminate the radical chain reactions and neutralize free radicals, which attack cells or biomolecules [17,18]. Moreover, antioxidants are additives used to protect food and pharmaceutical products against rancidity, unfavorable changes in color and structure, and extend shelf life by preventing unwanted odors. Additionally, some studies have demonstrated that synthetic antioxidants used in the foods and pharmaceuticals are toxic and may act as carcinogenic agents [19]. On the other hand, vegetables and fruits have a wide range of antioxidants and are a rich source of healthy food. In this regard, most antioxidant molecules obtained from natural sources such as plants have been found to be ROS or free radical scavengers [20,21]. For this reason, alternative, natural, and reliable plant-derived antioxidants are preferred as natural antioxidants [22,23].

Antioxidants delay or avoid the onset of major degenerative diseases including diabetes mellitus and Alzheimer’s disease (AD) [24,25,26]. One of the main targets in the treatment of diabetes is α-glycosidase whose activity is fundamental to the degradation of dietary polysaccharides. α-glycosidase inhibitors prevent the breakdown of polysaccharides into monosaccharide units and thus block the absorption of monomeric sugar units in the intestinal tract. In this way, it limits the postprandial plasma glucose level. α-glycosidase inhibitors can be used in the treatment of diabetes and as well as obesity [27,28].

AD is the most typical and common form of dementia among the older people that negatively affects the ability to perform personal daily activities. It is also well known that cholinergic conduction loss is one of the main causes of AD [29]. Therefore, acetylcholinesterase inhibitors (AChEIs) that enhance cholinergic transmission can be used for treatment of AD. Among them, tacrine is currently used in the palliative treatment for mild to moderate AD as AChEI. It is known that most of these drugs used today have undesired side effects including nausea, headache, vomiting, and diarrhea [30,31]. These clinical AChE inhibitors can exhibit undesired side effects including hepatotoxicity and gastrointestinal anomalies such as nausea and diarrhea [32,33]. Therefore, there is a great demand to develop and use AChEIs that are new and known for their antioxidant properties. With all this, phenolic compounds also have anti-AD properties and α-glycosidase inhibition profiles. Therefore, one of the most important approaches for treatment of neurodegenerative diseases and diabetes mellitus is natural antioxidant compounds and products [34,35,36,37]. However, current evidence suggests that patients with type-2 diabetes mellitus (T2DM) have an increased risk of developing AD. In addition, these evidences also show that hyperinsulinemia and insulin resistance-T2DM are distinguishing features [38,39].

Turkey has a rich plant biodiversity, and 11.6% of these plants are reported to be used for medicinal purposes to treat different diseases. Recently, there has been increasing focus and studies on endemic and medicinal plants for potential drug development and food preservative properties [40]. Additionally, it was reported that endemic plants of Turkey have shown a large spectrum of bioactivities [41]. The assessment of the bioactivity of kınkor (*Ferulago stellata*), an endemic plant of Turkey, has not been reported up to now. Therefore, the objective of this study was to evaluate the biological properties of different extracts (lyophilized water and evaporated ethanol extracts) of kınkor (*Ferulago stellata*). Moreover, biological properties of kınkor (*Ferulago stellata*) were determined by multiple bioanalytical antioxidant methods including Cu^2+^ reducing, Fe^3+^ reducing, and FRAP reducing abilities; DPPH˙ and ABTS˙^+^ scavenging activity; and Fe^2+^ chelating activity. In addition, another goal of the study was to determine its inhibition effect against some metabolic enzymes including α-glycosidase, α-amylase, and acetylcholinesterase, which are associated with Alzheimer’s disease and diabetes. In addition, characterization of chemical profile of both kınkor (*Ferulago stellata*) extracts was done by LC-HRMS.

## 2. Results and Discussion

**Figure 1:**
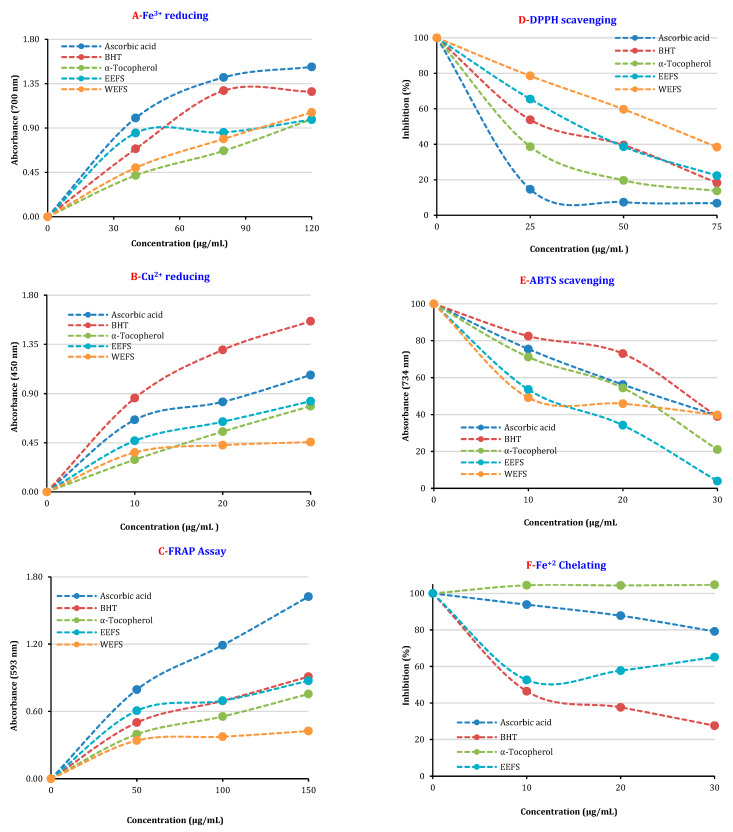
Fe^3+^ reducing (**A**), Cu^2+^ reducing (**B**), and Fe^3+^-TPTZ reducing (**C**); ABTS (**E**) and DPPH scavenging (**D**); and Fe^2+^ chelating (**F**) activities of EEFS and WEFS ((EEFS: evaporated ethanolic extract of aerial parts kınkor (*Ferulago stellata*); WEFS: lyophilized water extract of aerial parts of kınkor (*Ferulago stellata*) TPTZ: 2,4,6-tris(2-pyridyl)-s-triazine; DPPH: 1,1-diphenyl-2-picrylhydrazyl; ABTS: 2,2′-azino-bis(3-ethylbenzothiazoline-6-sulfonic acid)).

Antioxidant properties of ethanol and water extracts of kınkor (*Ferulago stellata*) have been carried out in different bioanalytical methods such as Fe^2+^ chelating activity, Fe^3+^ reducing activity, Fe^3+^-TPTZ reduction capacity, Cu^2+^ reduction ability, and ABTS and DPPH radicals scavenging activities. For comparison of antioxidant effects, putative standard compounds of α-tocopherol, ascorbic acid, and BHT were used for comparison. It was found that the antioxidant activities of ethanol and water extracts of kınkor (*Ferulago stellata*) are similar or close to used standard antioxidants. It was shown that the antioxidant activity of ethanol and water extracts of kınkor (*Ferulago stellata*) enhanced with increasing concentration (10–30 μg/mL). In some cases, the antioxidant ability of ethanol and water extracts of kınkor (*Ferulago stellata*) was observed to be higher than some standard antioxidants at the same concentration. In this context, reduction ability of ethanol and water extracts of kınkor (*Ferulago stellata*) enhanced with increasing concentration studied methods. It is well known that the reduction ability is one of the most significant factors in its total antioxidant effectiveness. The antioxidant activity of a molecule or extract can occur using different mechanisms [42,43]. Antioxidants may be in the form of stabilizing oxidants in redox reactions. The reduction capacity can be recorded by diverse bioanalytical methods. In the presence of reducing compounds, the reduction of (Fe[(CN)_6_]^3−^) to (Fe[(CN)_6_]^4−^) can easily occur. The addition of Fe^3+^ to the reduced product by addition of ethanol and water extracts of kınkor (*Ferulago stellata*) forms Fe_4_[Fe(CN)_6_], a complex in the Prussian blue color with sharp absorbance at 700 nm [44]. The enhanced absorbance shows the increased reduction capacity. The reducing capacity of ethanol and water extracts of kınkor (*Ferulago stellata*), BHT, α-tocopherol, and ascorbic acid increased constantly when the concentration of sample was increased. Fe^3+^ reducing capacity of ethanol and water extracts of kınkor (*Ferulago stellata*) and standards exhibited the following order: ascorbic acid (λ_700_: 1.520 ± 0.028, r^2^: 0.9970) > BHT (λ_700_: 1.269 ± 0.005, r^2^: 0.9880) > water extracts of kınkor (*Ferulago stellata*) (λ_700_: 1.058 ± 0.021, r^2^: 0.9973) > ethanol extracts of kınkor (*Ferulago stellata*) (λ_700_: 0.985 ± 0.013, r^2^: 0.9199) > α-tocopherol (λ_700_: 0.990 ± 0.007, r^2^: 0.9942) at 30 μg/mL. The results showed that ethanol and water extracts of kınkor (*Ferulago stellata*) had marked and powerful Fe^3+^ reducing effect (Figure 1A and Table 1). 

Another putative and commonly used method is Fe^3+^-TPTZ reduction assay [45]. The FRAP assay activity of ethanol and water extracts of kınkor (*Ferulago stellata*) declined in the following order (Figure 1C and Table 2): ascorbic acid (λ_593_: 1.624 ± 0.015, r^2^: 0.9930) > BHT (λ_593_: 0.909 ± 0.006, r^2^: 0.9874) > ethanol extract of kınkor (*Ferulago stellata*) (λ_593_: 0.873 ± 0.012, r^2^: 0.9553) > α-tocopherol (λ_593_: 0.755 ± 0.075, r^2^: 0.9867) > water extract of kınkor (*Ferulago stellata*) (λ_593_: 0.424 ± 0.016, r^2^: 0.9510) at 50 μg/mL. The FRAP method is carried out in an acidic environment to maintain iron solubility [46]. 

Copper is an important element in metallic form that can be found and used directly in nature. It is a very important cofactor for some endogenous and important metabolic enzymes including cytochrome c oxidase [47]. In addition, this chromogenic redox reaction is used to determine the potential of antioxidants containing non-protein thiols and thiols such as glutathione. Cupric ions (Cu^2+^) reducing power of same concentration (30 μg/mL) of ethanol and water extracts of kınkor (*Ferulago stellata*) and standards is shown in Table 1. A positive correlation was found between the Cu^2+^ reducing ability and different concentration of the ethanol and water extracts of kınkor (*Ferulago stellata*) (Figure 1B and Table 1). It was observed that Cu^2+^-reducing effect of ethanol and water extracts of kınkor (*Ferulago stellata*) increased with increasing concentrations (10–30 μg/mL). Cu^2+^-reducing ability of standards and both extracts at the same concentration (30 μg/mL) demonstrated the following order: BHT (λ_450_: 1.561 ± 0.089, r^2^: 0.9978) > ascorbic acid (λ_450_: 1.069 ± 0.007, r^2^: 0.9722) > α-tocopherol (λ_450_: 0.785 ± 0.061, r^2^: 0.9986) > ethanol extract of kınkor (*Ferulago stellata*) (λ_450_: 0.830 ± 0.022, r^2^: 0.9869) and water extract of kınkor (*Ferulago stellata*) (λ_450_: 0.456 ± 0.034, r^2^: 0.9742).

In the presence of O_2_ and transition metal ions, H_2_O_2_ can generate OH**^•^** via the Fenton reaction. In this way H_2_O_2_ is converted to a more reactive HO**^•^** by the Fenton reaction, which requires reduced iron ions (Fe^2+^), which had more reactivity than Fe^3+^ ions [48,49]. In this way, the formed OH radicals are more reactive than the end-peroxides. Metal binding effect of ethanol and water extracts of kınkor (*Ferulago stellata*) was evaluated using by two distinct metal chelator agents including ferrozine reagent. When the IC_50_ values of the binding effect of ethanol and water extracts of kınkor (*Ferulago stellata*) in the study were compared with the IC_50_ of the ethanol extract of kınkor (*Ferulago stellata*) and standard antioxidants, it was found to be as effective metal chelator with IC_50_: 31.5 ± 0.13 μg/mL (r^2^: 0.9030) (Figure 1F and Table 1) using ferrozine reagent, however, this value could not be detected for water extract of kınkor (*Ferulago stellata*). Additionally, relatively higher IC_50_ values were found for α-tocopherol (IC_50_: 33.0 ± 0.17 µg/mL, r^2^: 0.9109), ascorbic acid (IC_50_: 99.0 ± 0.36 μg/mL, r^2^: 0.9985), and BHT (IC_50_: 14.7 ± 0.56 μg/mL, r^2^: 0.9647).

The spectrophotometric methods based on the radical scavenging are frequently used to determine antioxidant abilities of pure substances, beverages, food, and herbal extracts. In addition, ABTS^∙+^ and DPPH∙ scavenging methods are fast, simple, selective and repeatable procedures. So, they are widely used to define the radical elimination abilities. It is easy to use the violet DPPH∙ and green-blue ABTS^∙+^ chromogens that have high sensitivity [50,51]. DPPH∙ scavenging method is mainly based on reduction of DPPH∙ that produces an easily identifiable strong violet color. The reduction of DPPH induces the radical to change violet to yellow color and this change is 517 nm [48,52]. As seen in Table 2, within the scope of DPPH∙ scavenging studies, IC_50_ values for ethanol and water extracts of kınkor (*Ferulago stellata*) had less effective DPPH∙ scavenging effect and were found to be 34.7 ± 0.22 μg/mL (r^2^: 0.9965) and 57.8 ± 0.07 μg/mL (r^2^: 0.9993), respectively when compared to α-tocopherol (23.1 ± 0.032 µg/mL, r^2^: 0.9825), ascorbic acid (16.1 ± 0.03 µg/mL, r^2^: 0.9566), and BHT (31.5 ± 0.01 µg/mL, r^2^: 0.9754), which are food additives used as preservative ingredients in some foods (Table 3 and Figure 1D). DPPH∙ scavenging assay is frequently used for detection of the antioxidant ability of pure compounds and plant extracts [53].

The formation of excessive free radicals in metabolism is one of the important factors that lead to the emergence of many chronic diseases [54]. As with DPPH radical scavenging ability, ABTS^∙+^ scavenging ability is extensively used for determination of radical scavenging activities of beverages, extracts, and pure substances [55]. ABTS^∙+^ is more reactive radical than DPPH radicals. As shown in Table 2, it was observed that ethanol and water extracts of kınkor (*Ferulago stellata*) had effective ABTS radical removing effects. The IC_50_ value of ABTS^∙+^ scavenging activity for ethanol and water extracts of kınkor (*Ferulago stellata*) was calculated as 7.8 ± 0.01 μg/mL (r^2^: 0.9844) and 19.3 ± 0.04 μg/mL (r^2^: 0.9419), respectively. Furthermore, this value was calculated as 26.7 ± 0.08 μg/mL (r^2^: 0.9717) for BHT, 15.4 ± 0.03 μg/mL (r^2^: 0.9825) for α-tocopherol, and 23.1 ± 0.01 μg/mL (r^2^: 0.9998) for ascorbic acid. The results clearly demonstrated that the ethanol and water extracts of kınkor (*Ferulago stellata*) have effective ABTS^∙+^ scavenging ability when compared to all standard antioxidants (Figure 1E and Table 2).

An important metabolic enzyme is acetylcholinesterase (AChE), which had been associated in some neurodegenerative diseases including AD [56]. The AChE inhibition had positive effect on the long-term progression of AD. In this context, there are many published studies on the inhibition potential of compounds and crude extracts. One such compound is galantamine and used to treat mild AD to moderate AD [57]. It is well known that natural products provide abundant and effective small molecule drug targets for the treatment of human diseases [58]. It is well known that phenolic antioxidants play an important role in avoiding or delaying the onset of major degenerative diseases, such as AD and T2DM [59]. In our study, we demonstrate that ethanol and water extracts of kınkor (*Ferulago stellata*) have a rich content of small molecules such as chlorogenic acid, rutin, and orientin. Additionally, ethanol extract of kınkor (*Ferulago stellata*) effectively inhibited AChE with IC_50_ values of 1.772 μg/mL (r^2^: 0.9831) for AChE. On the other hand, tacrine was used as positive control for AChE inhibition and had K_i_ value of 0.124 µM (r^2^: 0.9804) against AChE. AChE is the primary cholinesterase at mainly neuromuscular junctions and in chemical synapses in the body. However, it was observed that water extract of kınkor (*Ferulago stellata*) does not have any modulatory effects against the used metabolic enzymes.

Scientists have been extensively investigating the potential of medicinal plants to inhibit certain metabolic enzymes associated with some global diseases due to the various undesirable side effects of synthetic drugs. In this study, the ability of ethanol and water extracts of kınkor (*Ferulago stellata*) to modulate the activity of enzymes related to AD (AChE) and diabetes (α-glucosidase and α-amylase) was also investigated. Recently, diabetes is one of the fastest growing, serious, and costly health problems worldwide. A complete form of treatment and effective drugs for diabetes are still not found [60]. Plant extracts and their compounds have received great attention as antioxidants and potential inhibitors of key and metabolic enzymes, used in clinical conditions. For example, α-glycosidase and α-amylase enzymes, which serve as essential digestive enzymes in carbohydrate metabolism in the small intestine, have been considered targets and keys to reduce postprandial hyperglycemia (PPG) in diabetic patients [61]. In this context, important biologically active compounds such as acarbose, voglibose, and miglitol have been reported to reduce PPG by inhibiting α-glycosidase and α-amylase enzymes that perform carbohydrate digestion, thereby delaying or partially inhibiting glucose absorption from small intestines. Human saliva α-amylase is the most plentiful digestive enzyme in human saliva that hydrolyses polysaccharides such as starch to oligosaccharides [62]. Ethanol extract of kınkor (*Ferulago stellata*) had IC_50_ values of 0.826 μg/mL (r^2^: 0.9491) toward α-glycosidase and 0.639 μg/mL against α-amylase enzyme (r^2^: 0.9580). The results show that ethanol extract of kınkor (*Ferulago stellata*) as a crude extract exhibited efficient α-glycosidase and α-amylase inhibition effect when compared to acarbose as a starch blocker, which had IC_50_ of 10.00 μM for α-amylase and 22.80 μM for α-glycosidase [63].

The amount of total phenolic and flavonoids in medicinal plant extracts has been associated with their antioxidant capacity. Total phenolic compounds in ethanol and water extracts of kınkor (*Ferulago stellata*) were determined using the Folin-Ciocalteu reagent. Gallic acid, which is easily obtained in large amounts by acid or alkaline hydrolysis of tannin, was used for a standard graph (r^2^: 0.9840). Plants, vegetables, and fruits, which include polyphenols, are important sources of phenolic compounds in human diet. Accordingly, the consumption of foods containing phenolics, especially polyphenols, is of great importance in terms of natural antioxidants [64]. The quantity of phenolics in ethanol and water extracts of kınkor (*Ferulago stellata*) was determined using the equation taken from standard gallic acid graph and found as 31.36 and 56.36 gallic acid equivalents (GAE/mg extract), respectively. On the other hand, for determination of total flavonoids content of ethanol and water extracts of kınkor (*Ferulago stellata*), a standard gallic acid graphs was used. The flavonoids quantity in both extracts was determined as 35.98 and 28.50 μg quercetin equivalent (QE), respectively. The most favorable structural properties characterizing the antioxidative potential of phenolic compounds are the presence of hydrogen-donating substituents and the ability for delocalization of the resulting free electron for stability. The most active form of antioxidant molecules is the one that has more than one active group (e.g., -OH) in the ortho-position, which plays an important role in the structure-activity relationship of antioxidants [65]. It has been reported that the ortho-position is more active due to its ability to form intramolecular H-bonds, followed by the para-position and followed by then meta-position of the compounds. The H atom not involved in the intramolecular H-bond is then abstracted by free radicals, resulting in the formation of a stable molecule [66]. Plants rich in the specified compounds, therefore become a promising source of natural antioxidants. They are commercially grown and used in the pharmaceutical, food, and cosmetic industries. In addition, they are used not only as antioxidants but also as plants rich in many biological and biochemical applications [67].

Based on LC-HRMS analysis method, the most found phenolics identified in 1 mg of water extract of kınkor (*Ferulago stellata*) are rutin (14013.35 mg/kg), which is a naturally occurring flavonol glycoside in fruits, leafy vegetables, and several grains; chlorogenic acid as a polyphenolic compound that exhibits antioxidant, antibacterial, and antitumor activities (10103.18 mg/kg); and orientin that is a flavonoid from plant, derived often to use in various bioactivity studies (491.59 mg/kg). On the other hand, rutin that has wide variety of medicinal applications (156907.40 mg/kg); orientin (15329.03 mg/kg); and chlorogenic acid, as one of the natural products readily found in food, medicines, and cosmetics (44642.39 mg/kg), are the most plentiful phenolic compounds in 1 mg of ethanol extract of kınkor (*Ferulago stellata*) (Table 3). Different plant organs including fruits, vegetables, seeds, nuts, bark, and flowers are the main source of common natural phenolic compounds [68,69]. The antioxidant property of polyphenols from plants is well established. Phenolic compounds have biological functions including free radical scavenging and metal chelation, which prevent autoxidation. In plants, the antioxidant effects of phenolics are mainly due to redox effects. For this reason, hydrogen donors, reducing agents, singlet oxygen inhibitors, and metal chelates act as builders [70].

## 3. Materials and Methods

### 3.1. Chemicals and Plant Materials

α-tocopherol, neocuproine, DPPH radical, ABTS, DMPD, and α-tocopherol were obtained from Sigma-Aldrich (Stenheim, Germany). The sources and purity of the standard compounds for LC-HRMS are given as follows: ascorbic acid (≥99%, Sigma-Aldrich), (-)-epigallocatechin (>97%, TRC Canada, Toronto, Canada), (−)-epigallocatechin gallate (>97% TRC Canada), chlorogenic acid (≥95% Sigma-Aldrich), fumaric acid (≥99% Sigma-Aldrich), verbascoside (86.31%, HWI Analytik Gmbh, Rulzheim, Germany), orientin (>97%, TRC Canada), caffeic acid (≥98%, Sigma-Aldrich), (+)-trans taxifolin (>97%, TRC Canada), luteolin-7-rutinoside (>97%, Carbosynth limited, West Berkshire, UK), naringin (≥90%, Sigma-Aldrich), luteolin 7-glucoside (>97%, TRC Canada), rutin (≥94%, Sigma-Aldrich), rosmarinic acid (≥96%, Sigma-Aldrich), hyperoside (>97%, TRC Canada), dihydrokaempferol (>97%, Phytolab, Vestenbergsgreuth, Germany), quercitrin (>97%, TRC Canada), myricetin (>95%, Carl Roth GmbH + Co, Karlshue, Germany), quercetin (≥95%, Sigma-Aldrich), salicylic acid (≥98%, Sigma-Aldrich), naringenin (≥95%, Sigma-Aldrich), luteolin (95%, Sigma-Aldrich), nepetin (98%, Sigma-Aldrich), apigenin (>97%, TRC Canada), hispidulin (>97%, TRC Canada), isosakuranetin (>97%, Phytolab), CAPE (caffeic acid phenethyl ester) (≥97%, European Pharmacopoeia reference standard, Strasbourg, France), chrysin (≥96%, Sigma-Aldrich), acacetin (>97%, TRC Canada), and emodin (90%, Sigma-Aldrich). The other solvents used were of analytical grade and purchased from either Merck or Sigma-Aldrich. Kınkor (*Ferulago stellata*) was collected from B9 Van: Çatak, Bilgi village, surroundings of Üçüzler district, 2200 m, in August 2019 (location: 38°06′58.8″ N, 43°17′16.3″ E).

### 3.2. Preparation of the Water and Ethanol Extracts

The used water and ethanol extractions methods were previously described [71]. For determination of the ethanolic extract of aerial parts of kınkor (*Ferulago stellata*), a 50 g plant sample was cut into small pieces, ground into a fine powder using a mill and mixed with 0.5 L of ethyl alcohol, and then evaporated [72]. This process was repeated until the extraction solution turned colorless. The combined extracts were filtered through over Whatman paper and evaporated (Heidolph Hei-VAP HL, Germany). Dry ethanol extract of kınkor (*Ferulago stellata*) was transferred to an appropriate plastic bottle and kept at −20 °C until used in experiments.

For lyophilized water extraction shade-dried kınkor (*Ferulago stellata*), 50 g plant samples powdered and mixed with 500 mL water, boiled, and stirred for 20 min. Then extract was filtered and frozen at −87 °C in an ultra-low temperature freezer. Frozen extract was lyophilized at −50 °C at a pressure of 5 mm-Hg in a lyophilizator [73]. Prepared fresh lyophilized ethanolic extract of kınkor (*Ferulago stellata*) was kept in a plastic bottle and stored at −20 °C until used in experimental.

### 3.3. Reducing Ability Assays

The ferric ions (Fe^3+^) reducing ability of ethanol and water extracts of kınkor (*Ferulago stellata*) were realized according to Oyaizu [74] as given in previous literature [75,76]. Briefly, different concentrations of ethanol and water extracts of kınkor (*Ferulago stellata*) in distilled water or ethanol (10–50 µg/mL) were added to the same volume of phosphate buffers (1.25 mL, pH 6.6, 0.2 M) and K_3_Fe(CN)_6_ solution (1%, 1.25 mL). The mixtures were kept at 50 °C during 20 min and then, acidified with TCA (10%, 1.25 mL). Finally, a portion of FeCl_3_ (0.1%, 0.5 mL) was transferred and their absorbances were spectrophotometrically measured at 700 nm.

The Cu^2+^ ions reducing effects of ethanol and water extracts of kınkor (*Ferulago stellata*) were made according to spectrophotometric assay [77] as described in details [54]. For this aim, the same volumes of 250 μL of CuCl_2_ solution (10 mM, 0.25 mL), neocuproine solution (7.5 mM), and acetate buffer (0.25 mL, 1.0 M) were added to different concentrations of ethanol and water extracts of kınkor (*Ferulago stellata*) solutions (10–50 μg/mL) in test tubes. The volume of total mixture was adjusted to 2 mL with deionized water. Then, the tubes were closed and retained at 25 °C. Finally, their absorbances were spectrophotometrically recorded at 450 nm.

FRAP reduction ability was realized according to our previous study [78]. First, ethanol and water extracts of kınkor (*Ferulago stellata*) and standard solutions were transferred to the test tubes, which included several concentrations. A portion (2.25 mL) of TPTZ solution (10 mM TPTZ in 40 mM HCl) was freshly prepared and then transferred to 2.5 mL acetate buffer (0.3 M, pH 3.6) and 2.25 mL of FeCl_3_ solution (20 mM) in water. Then, different concentrations of ethanol and water extracts of kınkor (*Ferulago stellata*) (10–30 μg/mL) were dissolved in 5 mL of appropriate buffer solvent, stirred, and incubated at 37 °C for 30 min. Finally, the absorbance of mixture was spectrophotometrically measured at 593 nm.

### 3.4. Radical Scavenging Activities

DPPH∙scavenging ability of ethanol and water extracts of kınkor (*Ferulago stellata*) was performed according to Blois method [79] as given prior studies [80,81,82]. DPPH radicals were used for the estimation of the radical scavenging capacity of plant extracts. In brief, an aliquot of DPPH radicals (0.5 mL, 0.1 mM) was added to ethanol and water extracts of kınkor (*Ferulago stellata*) solution (1.5 mL) in ethanol or water (10–50 µg/mL) and incubated for 30 min in the dark. Finally, the absorbance of the mixtures was spectrophotometrically recorded at 517 nm.

ABTS^∙+^ scavenging ability of ethanol and water extracts of kınkor (*Ferulago stellata*) was realized according to the previous study [83,84]. Primarily an ABTS cation radical solution (7.0 mM) was produced by adding K_2_S_2_O_8_ to an ABTS solution and their absorbances was set to 0.750 ± 0.025 nm diluted by buffer solution at 734 nm. Finally, 3.0 mL of ethanol and water extracts of kınkor (*Ferulago stellata*) at various concentrations (10–50 μg/mL) were mixed with 1.0 mL of ABTS^•+^ and the remaining absorbance was spectrophotometrically recorded at 734 nm.

The radical removing capacities (RRC) of ethanol and water extracts of kınkor (*Ferulago stellata*) were found as millimolar in the reaction medium. Both radicals (DPPH^•^ and ABTS^•+^) scavenging effects were calculated as follows:

RRC (%) = (1 − A_Sample_/A_Control_) × 100, (1)

where A_Control_ and A_Sample_ are the absorbance values of the control and samples, respectively. The half maximal inhibitory concentration (IC_50_) was estimated by plotting percentages against the ethanol and water extracts of kınkor (*Ferulago stellata*) sample concentrations (μg/mL) [85,86].

### 3.5. Anticholinergic Assay

AChE and BChE inhibitions of ethanol and water extracts of kınkor (*Ferulago stellata*) are used within the scope of anticholinergic studies. The AChE inhibitory effect of ethanol and water extracts of kınkor (*Ferulago stellata*) was realized according to Ellman’s method [87] as given in previous studies [88,89]. AChE was obtained from electric eel (*Electrophorus electricus*). For this, 5,5′-dithio-bis-(2-nitrobenzoic acid) (DTNB) and acetylthiocholine iodide (AChI) were used as substrate for cholinergic reaction [90]. Briefly, 100 μL of Tris/HCl buffer (1.0 M, pH 8.0) and different concentrations of ethanol and water extracts of kınkor (*Ferulago stellata*) solution were dissolved in ethanol and deionized water. Then, 50 μL of AChE (5.32 × 10^−3^ EU) solution was added and incubated for 10 min at 25 °C. After a short incubation period, 50 μL of DTNB (0.5 mM) was added. Finally, the reaction was started by the addition of 50 μL of acetylcholine iodate (AChI) (10 mM). The enzymatic hydrolysis of these substrates was spectrophotometrically determined by the formation of yellow 5-thio-2-nitrobenzoate anion as the result of the reaction of DTNB with thiocholine at a wavelength of 412 nm.

### 3.6. Antidiabetic Assay 

Two digestive enzyme inhibitions of ethanol and water extracts of kınkor (*Ferulago stellata*) were studied within the scope of the antidiabetic study. α-glycosidase inhibition efficacy of ethanol and water extracts of kınkor (*Ferulago stellata*) was performed according to Tao et al. [91] using p-nitrophenyl-d-glycopyranoside (p-NPG) substrate as described previously in details. The absorbances of samples were spectrophotometrically recorded at 405 nm [92]. First, 75 μL of phosphate buffer was mixed with 20 μL of the α-glycosidase solution (0.15 U/mL) in phosphate buffer (0.15 U/mL) pH 7.4) and 5 μL of different concentration ethanol and water extracts of kınkor (*Ferulago stellata*) sample dissolved in ethanol and deionized water, respectively. Then, it was pre-incubated at 35 °C for 10 min prior to the addition of p-NPG to the initiation of the reaction. In addition, 20 μL of p-NPG was added in phosphate buffer (5 mM, pH 7.4) after re-incubation at 35 °C. The absorbances were spectrophotometrically measured at 405 nm.

α-Amylase activity, the second digestive enzyme, was determined according to the Xiao’s procedure [93]. Starch was used as substrate and dissolved in 80 mL NaOH solution (0.4 M, 30 min, 80 °C). For this, 35 μL of starch solution, 35 μL of phosphate buffer (pH 6.9), and 10 μL of different concentrations of ethanol and water extracts of kınkor (*Ferulago stellata*) sample dissolved in ethanol and deionized water were mixed and was preincubated at 35 °C for 30 min. Then, 20 μL of α-amylase solution was added to it and incubated for 30 min. The reaction was finished by addition of 50 μL of HCl (0.1 M). The absorbances were spectrophotometrically measured at 580 nm.

### 3.7. Determination of Inhibition Parameters

The IC_50_ was obtained from activity (%) versus ethanol and water extracts of kınkor (*Ferulago stellata*) concentration plots. Furthermore, Lineweaver-Burk [94] graphs were used for determination of K_i_ and other inhibition types [95].

### 3.8. Total Phenolic and Flavonoid Contents

Total phenolics in ethanol and water extracts of kınkor (*Ferulago stellata*) were calculated by Folin-Ciocalteu methods [96] as descried in prior studies [97]. The results were calculated as μg of gallic acid equivalents (GAE) per g of extract (μg GAE/g). The amount of total phenolics in ethanol and water extracts of kınkor (*Ferulago stellata*) were calculated from the calibration curve. Total flavonoids in ethanol and water extracts of kınkor (*Ferulago stellata*) were determined according to our previous colorimetric method [98]. The aluminum chloride (AlCl_3_) colorimetric assay was used for the estimation of the total flavonoid content. The standard quercetin curve (0–100 μg/mL) was used to determine total flavonoids, and results are given as μg quercetin equivalents (QE) per g ethanol and water extracts of kınkor (*Ferulago stellata*).

### 3.9. Preparation of Samples for LC-HRMS Analysis

The dried 100 mg of the ethanol and water extracts of kınkor (*Ferulago stellata*) were dissolved in water in a 5 mL volumetric flask, which was kept in an ultrasonic bath until a clear solution was obtained. Then, 0.1 mL of dihydrocapsaicin solution, used as an internal standard, was added and diluted to the volume with mobile phase and stirred and heated to get clear solution. Then, the solution was filtered (0.45 µm Millipore Millex-HV filter). The concentration of final solution (1 mL) was added in a capped auto sampler vial, from which 2 µL of sample was injected to LC for each run. The prepared samples in the auto sampler were stored at 15 °C [99,100,101].

### 3.10. Instruments and Chromatographic Conditions of LC-HRMS

LC-HRMS experiments were performed on a Thermo ORBITRAP Q-EXACTIVE mass spectrometry (Bremen, Germany) equipped with a Troyasil C18 column (150 mm × 3 mm i.d., 3 µm particle size, Istanbul, Turkey). The mobile phases A and B were composed of 1% formic acid–water and 1% formic acid–methanol, respectively. The gradient program of which was 0–1.00 min 50% A and 50% B, 1.01–6.00 min 100% B, and finally 6.01–10 min 50% A and 50% B. The flow rate of the mobile phase was 0.35 mL/min, and the column temperature was set to 22 °C. Environmental conditions were set as temperature of 22.0 ± 5.0 °C and relative humidity of (50 ± 15)% rh [102,103].

### 3.11. Optimization of LC-HRMS Procedure

**Figure 2:**
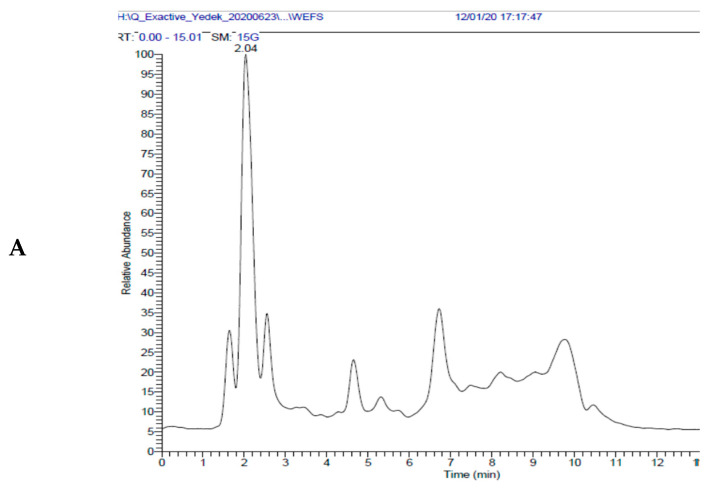

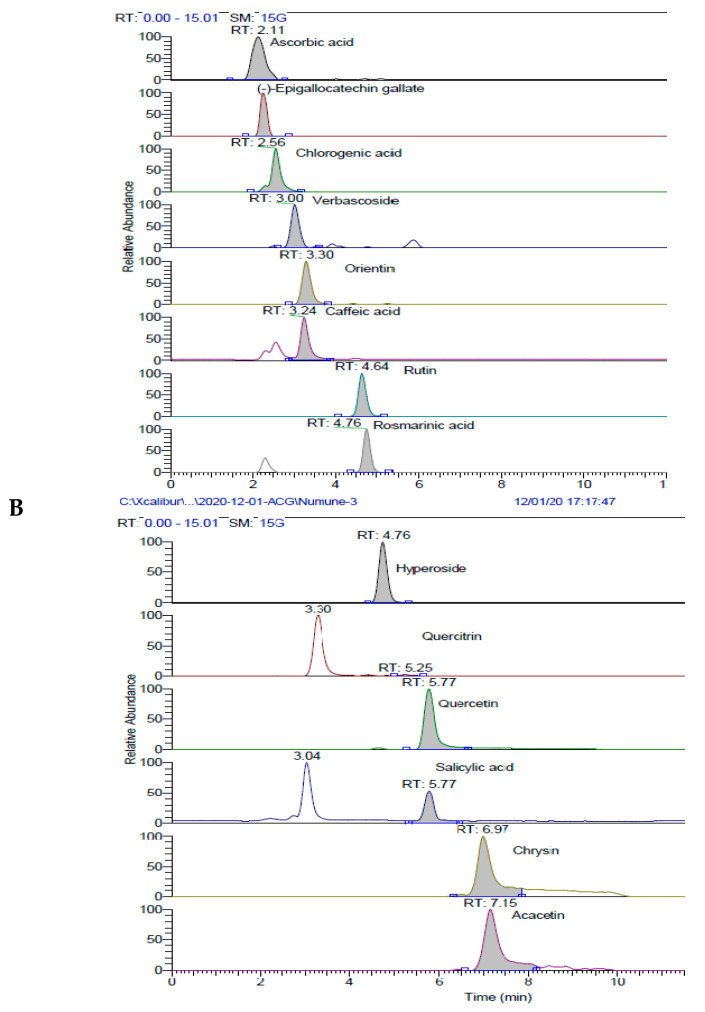
Liquid chromatography-high-resolution mass spectrometry (LC-HRMS) chromatogram of WEFS measurements (WEFS: lyophilized water extract of aerial parts of kınkor (*Ferulago stellata*)). (**A**) Total ion chromatogram (TIC) in negative ionization mode and (**B**) HRMS.

The best mobile phase was found to be an acidified methanol and water gradient in HPLC method. This mobile phase has also been found to be suitable for ionization abundance and separation of compounds. The best ionization of small and relatively polar compounds has been achieved with the ESI source. The ions between *m*/*z* 85–1500 were scanned in high-resolution mode of instrument [104,105]. Identification of the compounds was accomplished by comparing the retention times of the standard compounds (in the purity range of 95–99%; see section chemicals) and HRMS data of Bezmialem Vakif University, Drug Application and Research Center Library (ILMER). Dihydrocapsaicin (purity 95%) was used as an internal standard in LC-HRMS measurements to reduce the repeatability problem caused by external influences such as ionization repeatability in mass spectrometry measurements. TIC chromatogram of EESF and WESF in negative and positive ionization modes were given in Supplementary Materials (Figures S1–S4). The detailed mass parameters of each target compounds are given in Figure 2 and Table 4.

## 4. Conclusions

Data presented in this study demonstrated that kınkor (*Ferulago stellata*), an understudied endemic plant to Turkey, possessed effective antioxidant and some metabolic enzymes inhibitory properties. Evaluation of bioactivity and phytochemical screening of kınkor (*Ferulago stellata*) had great importance. Ethanol and water extracts of kınkor (*Ferulago stellata*) have been found to have efficient antioxidant properties when compared to BHA, BHT and ascorbic acid in various bioanalytical tests, including Fe^3+^ and Cu^2+^ reduction abilities, Fe^2+^ binding, as well as DPPH and ABTS radical scavenging activities. In addition, ethanol extract possessed higher antioxidant activity, phenolic contents, and demonstrated AChE, α-glycosidase and α-amylase inhibition effects. This study suggests that ethanol and water extracts of kınkor (*Ferulago stellata*) could be a promising potential source of beneficial phenolics. 

## Figures and Tables

**Table 1:** The reducing power of the EEFS and WEFS and standards antioxidants by Fe^3+^ reducing (120 μg/mL), Cu^2+^ reducing (30 μg/mL), and Fe^3+^-TPTZ reducing (150 μg/mL) methods ((EEFS: evaporated ethanolic extract of aerial parts kınkor (*Ferulago stellata*); WEFS: lyophilized water extract of aerial parts of kınkor (*Ferulago stellata*); TPTZ: 2,4,6-tris(2-pyridyl)-s-triazine).

Antioxidants	Fe^3+^ Reducing		Cu^2+^ Reducing		Fe^3+^-TPTZ Reducing	
	λ_700_	r^2^	λ_450_	r^2^	λ_593_	r^2^
α-tocopherol	0.990 ± 0.007	0.9942	0.785 ± 0.061	0.9986	0.755 ± 0.075	0.9867
Ascorbic acid	1.520 ± 0.028	0.9970	1.069 ± 0.007	0.9722	1.624 ± 0.015	0.9930
BHT	1.269 ± 0.005	0.9880	1.561 ± 0.089	0.9978	0.909 ± 0.006	0.9874
EEFS	0.985 ± 0.013	0.9199	0.830 ± 0.022	0.9869	0.873 ± 0.012	0.9553
WEFS	1.058 ± 0.021	0.9973	0.456 ± 0.034	0.9742	0.424 ± 0.016	0.9510

**Table 2:** The half maximum concentration (IC_50_, μg/mL) of EEFS, WEFS, and standards for the DPPH and ABTS radicals scavenging activities and ferrous ion chelating ability (EEFS: evaporated ethanolic extract of aerial parts kınkor (*Ferulago stellata*); WEFS: lyophilized water extract of aerial parts of kınkor (*Ferulago stellata*)).

Compounds	DPPH^•^ Scavenging		ABTS^•+^ Scavenging		Fe^2+^ Chelating	
	IC_50_ *	r^2^	IC_50_ *	r^2^	IC_50_ *	r^2^
α-tocopherol	23.1 ± 0.032	0.9825	15.4 ± 0.03	0.9866	33.0 ± 0.17	0.9109
Ascorbic acid	16.1 ± 0.03	0.9566	23.1 ± 0.01	0.9998	99.0 ± 0.36	0.9985
BHT	31.5 ± 0.01	0.9754	26.7 ± 0.08	0.9717	14.8 ± 0.56	0.9646
EEFS	34.7 ± 0.22	0.9965	7.8 ± 0.01	0.9844	31.5 ± 0.13	0.903
WEFS	57.8 ± 0.07	0.9993	19.3 ± 0.04	0.9419	- *	- *

* They were not determined.

**Table 3:** Liquid chromatography-high-resolution mass spectrometry (LC-HRMS) analysis of EEFS and WEFS (EEFS: evaporated ethanolic extract of aerial parts kınkor (*Ferulago stellata*); WEFS: lyophilized water extract of aerial parts of kınkor (*Ferulago stellata*)).

No	Compounds	WEFS	EEFS	U (%)
1	Ascorbic acid	47.41	172.44	3.94
2	(−)-Epigallocatechin	<LOD	<LOD	3.09
3	(−)-Epigallocatechin gallate	1.59	<LOD	3.76
4	Chlorogenic acid	10103.18	44642.39	3.58
5	Fumaric acid	<LOD	3109.11	2.88
6	Verbascoside	6.59	225.72	2.93
7	Orientin	491.59	15329.03	3.67
8	Caffeic acid	24.41	126.39	3.74
9	(+)-trans taxifolin	<LOD	2.10	3.35
10	Luteolin-7-rutinoside	<LOD	<LOD	3.06
11	Naringin	<LOD	<LOD	4.20
12	Luteolin 7-glucoside	<LOD	<LOD	4.14
13	Rutin	14013.35	156907.40	3.07
14	Rosmarinic acid	26.88	134.22	3.77
15	Hyperoside	105.94	2633.75	3.46
16	Dihydrokaempferol	<LOD	2.33	2.86
17	Quercitrin	3.82	105.78	3.78
18	Myricetin	<LOD	0.47	4.18
19	Quercetin	30.82	197.18	2.95
20	Salicylic acid	27.53	130.76	1.89
21	Naringenin	<LOD	23.12	4.20
22	Luteolin	<LOD	11.06	3.42
23	Nepetin	<LOD	<LOD	2.19
24	Apigenin	<LOD	8.60	2.87
25	Hispidulin	<LOD	66.12	3.41
26	Isosakuranetin	<LOD	<LOD	3.98
27	Caffeic acid phenethyl ester	<LOD	0.23	3.13
28	Chrysin	6.47	1.38	3.24
29	Acacetin	5.53	9.92	3.98
30	Emodin	<LOD	1.56	4.27

**Table 4:** LC-HRMS method parameters of selected compounds in EEFS and WEFS (EEFS: evaporated ethanolic extract of aerial parts kınkor (*Ferulago stellata*); WEFS: lyophilized water extract of aerial parts of kınkor (*Ferulago stellata*)).

Compounds	RT	*m*/*z*	δ ppm	Ionization Mode	Linear Range	Linear Regression Equation	LOD/LOQ	R^2^	Recovery
Ascorbic acid	1.99	175.0248	−0.81	Negative	0.5–10	y = 0.00347x − 0.00137	0.39/1.29	0.9988	96.20
(−)-Epigallocatechin	2.15	307.0812	−1.07	Positive	0.3–5	y = 0.00317x + 0.000443	0.17/0.57	0.9947	102.22
Chlorogenic acid	2.21	353.0878	−0.91	Negative	0.05–10	y = 0.00817x + 0.000163	0.02/0.06	0.9994	96.68
Verbascoside	2.43	623.1981	−0.61	Negative	0.1–10	y = 0.00758x + 0.000563	0.03/0.1	0.9995	96.19
Orientin	2.45	447.0933	−0.45	Negative	0.1–10	y = 0.00757x + 0.000347	0.01/0.03	0.9993	96.22
Caffeic acid	2.89	179.0350	1.72	Negative	0.3–10	y = 0.0304x + 0.00366	0.08/0.27	0.9993	94.51
Luteolin-7-rutinoside	3.09	593.1512	−0.26	Negative	0.1–10	y = 0.00879x + 0.000739	0.01/0.03	0.9988	93.05
Naringin	3.17	579.1719	−0.07	Negative	0.05–10	y = 0.00576x − 0.000284	0.01/0.03	0.9991	101.91
Luteolin 7-glucoside	3.85	447.0933	−0.32	Negative	0.1–7	y = 0.0162x + 0.00226	0.01/0.03	0.9961	96.31
Hesperidin	3.85	609.1825	0.29	Negative	0.05–10	y = 0.00423x + 0.0000138	0.01/0.03	0.9994	96.14
Rutin	4.12	609.1461	0.12	Negative	0.05–10	y = 0.00329x − 0.00005576	0.01/0.03	0.999	96.97
Syringic acid	4.24	197.0456	−0.26	Negative	0.5–10	y = 0.0000831x + 0.000024	0.1/0.3	0.9991	97.29
Rosmarinic acid	4.48	359.0772	0.01	Negative	0.05–10	y = 0.00717x − 0.0003067	0.01/0.03	0.9992	99.85
Hyperoside	4.66	463.0882	−0.17	Negative	0.05–10	y = 0.0072x − 0.00003096	0.01/0.03	0.9995	96.62
Apigenin 7-glucoside	4.58	431.0984	−0.06	Negative	0.3–7	y = 0.0246x + 0.00306	0.01/0.03	0.9962	96.07
Quercitrin	4.88	447.0933	−0.18	Negative	0.05–10	y = 0.0179 + 0.0003331	0.01/0.03	0.999	97.00
Quercetin	5.13	301.0354	−0.32	Negative	0.1–10	y = 0.0509x + 0.00467	0.01/0.03	0.9978	96.41
Salicylic acid	5.15	137.0244	−0.44	Negative	0.3–10	y= 0.0361x + 0.00245	0.01/0.03	0.9982	92.88
Naringenin	5.68	271.0612	−0.12	Negative	0.1–10	y = 0.0281x + 0.00182	0.01/0.03	0.9995	86.65
Luteolin	5.72	285.0405	0.46	Negative	0.1–10	y = 0.117x + 0.00848	0.01/0.03	0.9981	96.98
Apigenin	5.74	269.0456	−0.25	Negative	0.3–10	y = 0.104x + 0.0199	0.01/0.03	0.9998	81.55
Hispidulin	5.84	301.0707	−0.18	Positive	0.05–10	y = 0.02614x + 0.0003114	0.01/0.03	0.9993	98.36
Isosakuranetin	5.86	285.0769	−0.21	Negative	0.05–10	y = 0.0235x + 0.000561	0.01/0.03	0.9992	96.56
Chrysin	6.20	253.0506	−0.29	Negative	0.05–7	y = 0.0964x − 0.0002622	0.01/0.03	0.999	87.92
Acacetin	6.24	283.0612	−1.08	Negative	0.05–7	y = 0.046x + 0.0001875	0.01/0.03	0.9995	87.52

## Data Availability

Data available in a publicly accessible repository.

## Supplementary Materials

The following are available online. Figure S1: TIC chromatogram of EESF in negative ionization mode, Figure S2: TIC chromatogram of EESF in pozitive ionization mode, Figure S3: TIC chromatogram of WESF in negative ionization mode, Figure S4: TIC chromatogram of WESF in pozitive ionization mode.

## Sample Availability

Samples of the compounds are not available from the authors.
