# Supplementary material for: LC-HRMS Profiling and Antidiabetic, Anticholinergic, and Antioxidant Activities of Aerial Parts of Kınkor (Ferulago stellata)
Source: Molecules. 2021 Apr 23;26(9):2469. doi: 10.3390/molecules26092469 (PMC8122897; doi:10.3390/molecules26092469)
Supplement: Supplementary file 1 [file molecules-26-02469-s001.zip › molecules-1160582-supplementary.pdf]

Supporting Information

LC-HRMS Profiling, Antidiabetic, Anticholinergic and Antioxidant Activities of Aerial Parts of Kınkor (*Ferulago stellata*)

Hatice Kızıltaş [1], Zeynebe Bingol [2,3], Ahmet Ceyhan Gören [4,5], Leyla Polat Kose [6], Lokman Durmaz [7], Fevzi Topal [8], Saleh H. Alwasel [9] and İlhami Gulcin [2]

[1] Department of Pharmacy Services, Vocational School of Health Services, Van Yuzuncu Yil University, 65080-Van, Turkey
[2] Department of Chemistry, Faculty of Science, Ataturk University, Erzurum, Turkey
[3] Department of Medical Services and Techniques, Tokat Vocational School of Health Services, Gaziosmanpasa University, 60250-Tokat, Turkey
[4] Department of Analytical Chemistry, Faculty of Pharmacy, Bezmialem Vakif University, 34093-Istanbul, Turkey
[5] Drug Application and Research Center, Bezmialem Vakif University, 34093-Istanbul, Turkey
[6] Department of Pharmacy Services, Vocational School, Beykent University, 34500-Buyukcekmece, Istanbul, Turkey
[7] Department of Medical Services and Technology, Cayirli Vocational School, Erzincan Binali Yildirim University, 24500-Cayirli, Erzincan, Turkey
[8] Department of Chemical and Chemical Processing Technologies, Gumushane Vocational School, Gumushane University, 29000-Gumushane, Turkey
[9] Department of Zoology, College of Science, King Saud University, 11451-Riyadh, Saudi Arabia

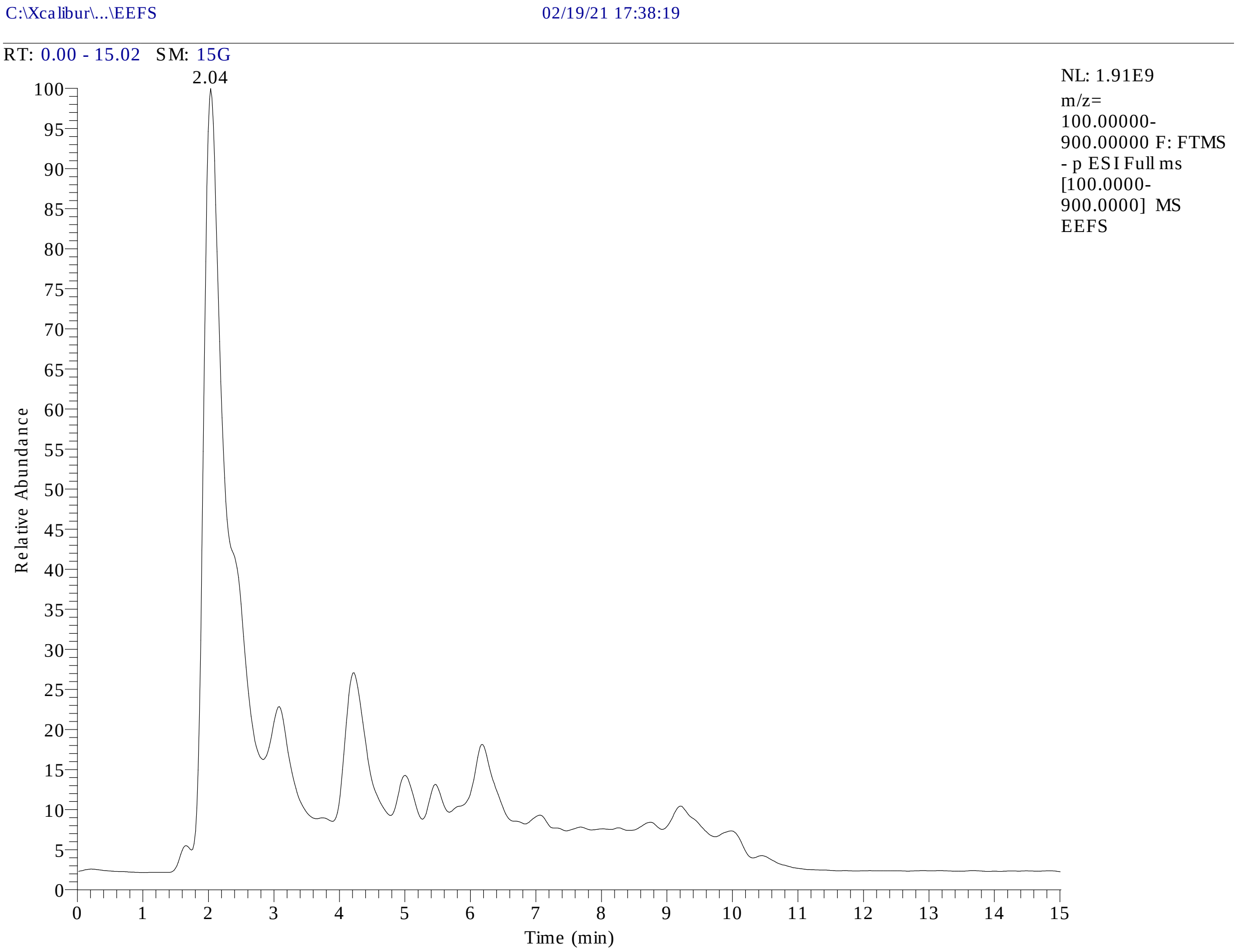

Figure S1. TIC chromatogram of EESF in negative ionization mode.

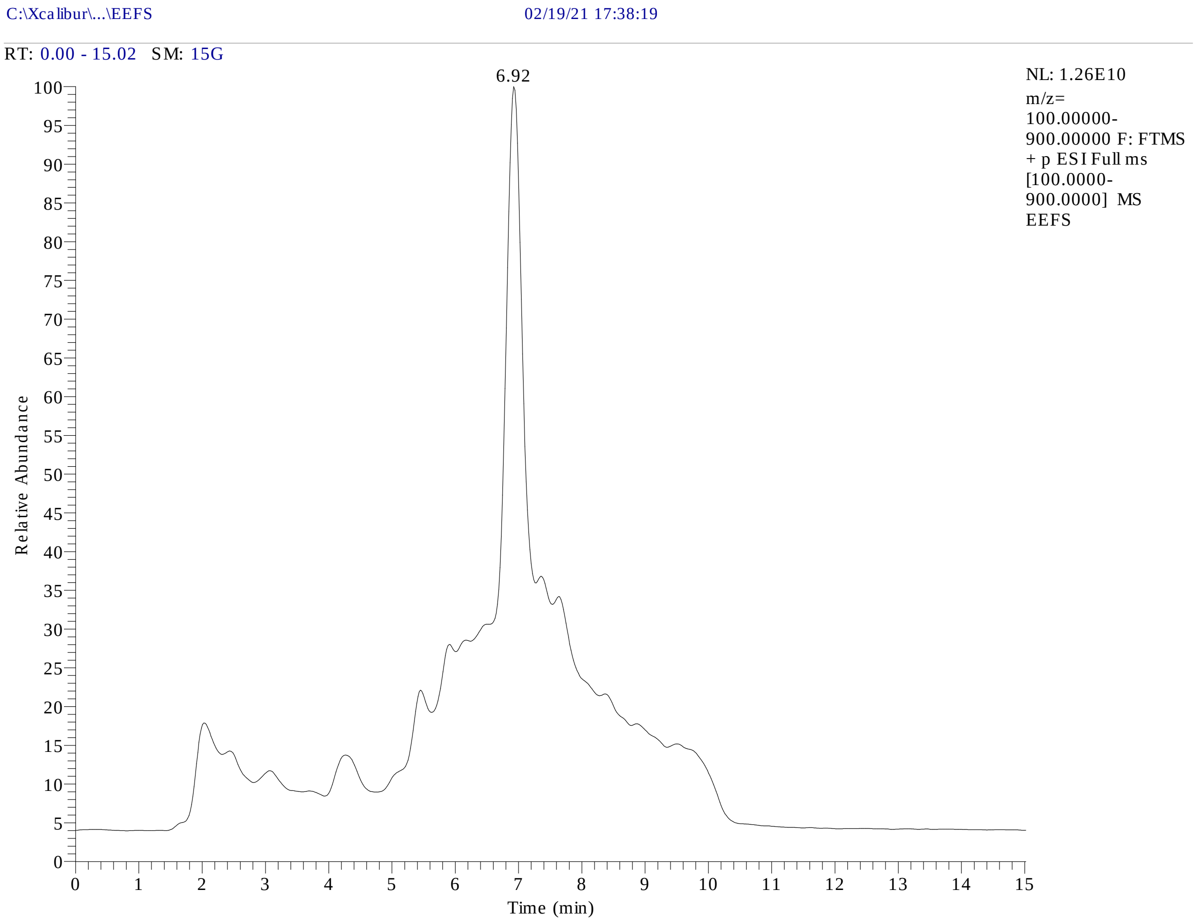

Figure S2. TIC chromatogram of EESF in pozitive ionization mode.

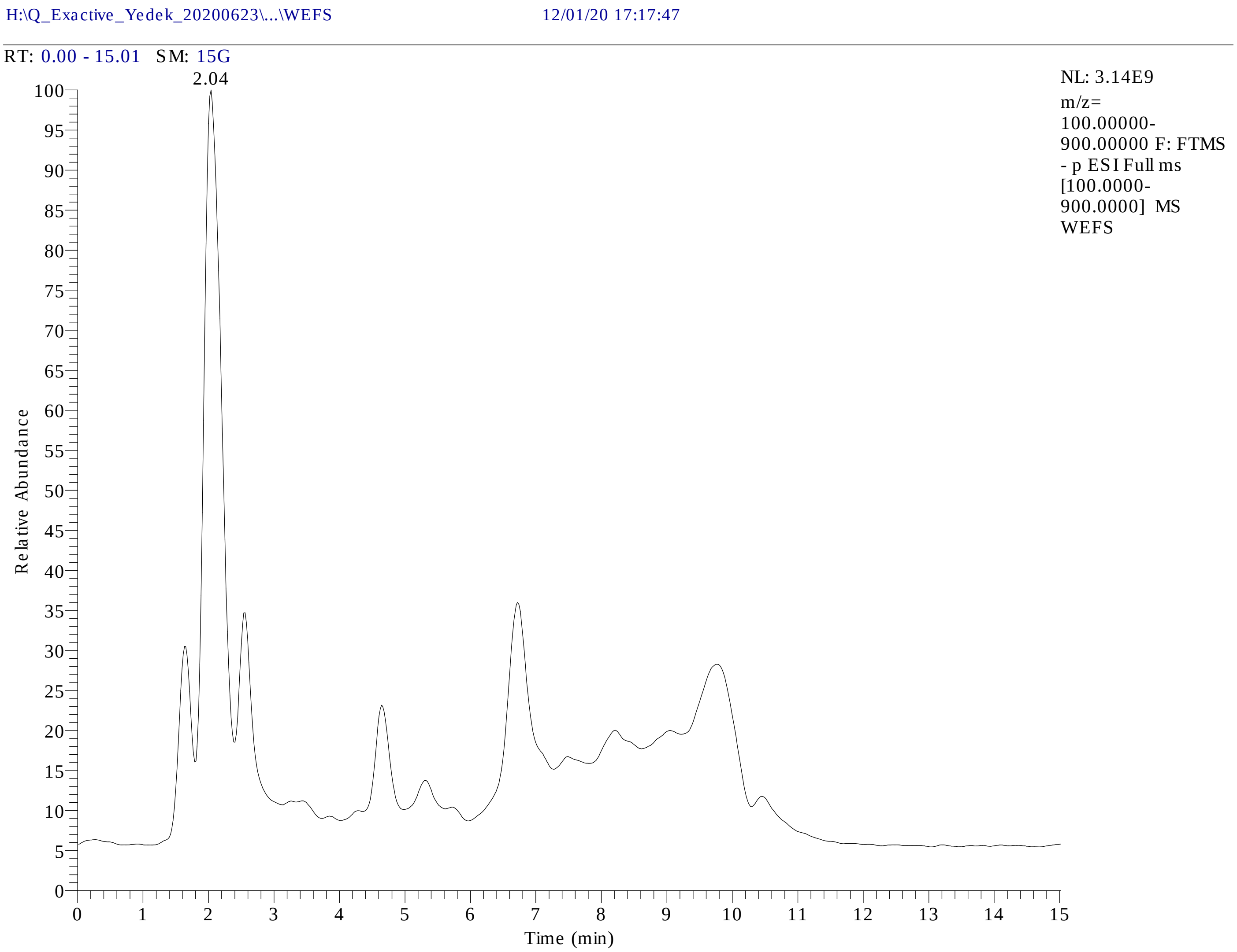

Figure S3. TIC chromatogram of WESF in negative ionization mode.

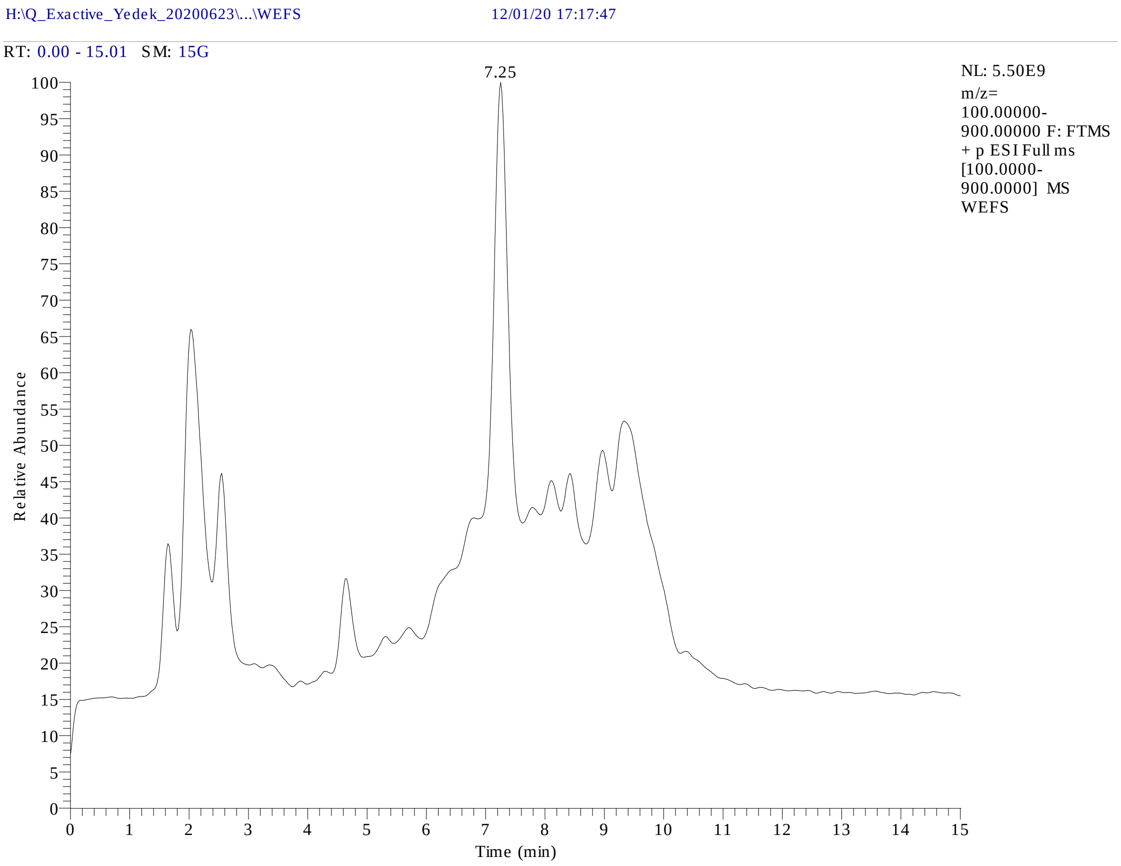

Figure S4. TIC chromatogram of WESF in pozitive ionization mode.
